# Extracellular Vesicles in Adipose Tissue Communication with the Healthy and Pathological Heart

**DOI:** 10.3390/ijms24097745

**Published:** 2023-04-24

**Authors:** Lauriane Y. M. Michel

**Affiliations:** Pole of Pharmacology and Therapeutics, Institut de Recherche Expérimentale et Clinique (IREC), Université Catholique de Louvain (UCLouvain), 57 Avenue Hippocrate, 1200 Brussels, Belgium; lauriane.michel@uclouvain.be; Tel.: +32-2-764-52-62

**Keywords:** adipose tissue, extracellular vesicles, exosomes, heart disease, heart failure, endocrine communication, adipocytes, obesity, cardiac physiology, ischemia

## Abstract

Adipose tissue and its diverse cell types constitute one of the largest endocrine organs. With multiple depot locations, adipose tissue plays an important regulatory role through paracrine and endocrine communication, particularly through the secretion of a wide range of bioactive molecules, such as nucleic acids, proteins, lipids or adipocytokines. Over the past several years, research has uncovered a myriad of interorgan communication signals mediated by small lipid-derived nanovesicles known as extracellular vesicles (EVs), in which secreted bioactive molecules are stably transported as cargo molecules and delivered to adjacent cells or remote organs. EVs constitute an essential part of the human adipose secretome, and there is a growing body of evidence showing the crucial implications of adipose-derived EVs in the regulation of heart function and its adaptative capacity. The adipose tissue modifications and dysfunction observed in obesity and aging tremendously affect the adipose-EV secretome, with important consequences for the myocardium. The present review presents a comprehensive analysis of the findings in this novel area of research, reports the key roles played by adipose-derived EVs in interorgan cross-talk with the heart and discusses their implications in physiological and pathological conditions affecting adipose tissue and/or the heart (pressure overload, ischemia, diabetic cardiomyopathy, etc.).

## 1. Introduction

Heart diseases remain a leading cause of morbidity and mortality despite substantial advances in prevention and treatment [1]. The endocrine action of adipose tissue is not a recent finding [2]. Since the initial discovery of its capacity to modulate appetite, it has been demonstrated that adipose tissue is in fact implicated in the regulation of many biological functions by secreting a vast array of factors, including hormones, adipokines, lipids and nucleic acids. The importance of the adipose tissue secretome in cardiovascular health and disease remains the focus of extensive research. In the last several years, an additional actor of the adipose secretome has been brought to light: extracellular vesicles (EVs). These small lipid-derived nanovesicles, which are released by all cell types but particularly by adipose tissues, can stably deliver secreted bioactive factors to adjacent cells or remote organs. The study of adipose-derived EVs in the past several years has further expanded our knowledge of the role of adipose tissue communication in cardiac physiology and its impact on heart disease pathogenesis. In this review, we present the findings in this novel area of research and highlight the implications of adipose-derived EVs on cardiac function and remodeling. First, we review the heterogeneity of adipose tissue and the metabolic functions carried out by diverse adipose depots as well as their endocrine actions in cardiovascular settings. Next, we review the biogenesis of extracellular vesicles in adipose tissue, their classification and their capacity for cross-talk. We then discuss their implications for the heart depending on adipose tissue diversity and remodeling, with a particular emphasis on EVs released from white or brown adipocytes and the modified cross-talk in dysfunctional adipose tissue. Finally, we highlight the different types of modulations induced by adipose-derived EVs during heart disease pathogenesis (pressure overload, ischemia, diabetic cardiomyopathy, atrial fibrillation, etc.).

## 2. Adipose-Tissue-Derived Extracellular Vesicles

### 2.1. Adipose Tissue Diversity

#### 2.1.1. Multiple and Diverse Adipose Depots

**Figure 1:**
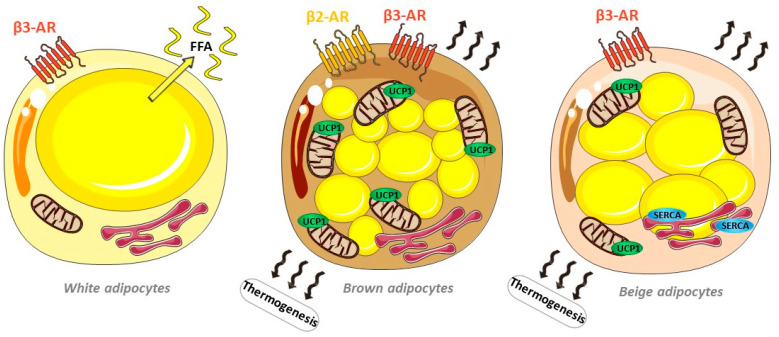
Schematic representation of white, beige and brown adipocytes and their thermogenesis mechanisms, including activation of the lipolysis of lipid droplets via beta-adrenergic stimulation, the uncoupling of mitochondrial respiration via uncoupling protein 1 (UCP1) and futile Ca^2+^ cycling via the endoplasmic reticulum Ca^2+^ ATPase SERCA. In white adipocytes, beta-adrenergic stimulation induces lipolysis, leading to non-esterified free fatty acid release from their unique lipid droplets in the absence of thermogenesis.

Adipose tissue can be broadly divided into two types of tissue: white and brown adipose tissues. White adipose tissue (WAT) represents most of the adipose tissue mass in the human body. WAT stores excess energy in the form of triglycerides within a single lipid droplet and supplies fuel to peripheral tissues under conditions of a negative energy balance through the regulated release of free fatty acids. WAT depots are mainly located either intra-abdominally/intra-thoracically (e.g., visceral adipose tissue (VAT) and surrounding organs such as mesenteric, perirenal, omental, epicardial and gonadal depots) or subcutaneously (SAT) (such as abdominal, gluteo-femoral, inguinal and subscapular depots). VAT is considered more detrimental to overall health and correlates with an elevated risk of diabetes, hypertension and atherosclerosis [3,4] and was recently associated with cardiac aging [5]. WAT comprises heterogeneous cell types (adipocytes, adipocyte precursors, immune cells, fibroblasts, endothelial cells, etc.) with cellular proportions varying among adipose depots and metabolic statuses. In obesogenic conditions with an excess of nutrients, white adipocytes adapt using two processes, namely, hyperplasia (cell proliferation) and hypertrophy (cell enlargement), which are associated with a heightened state of inflammation due to the infiltration of immune cells, particularly macrophages within the depots. Under conditions of a negative energy balance, sympathetic noradrenaline released and delivered to white adipocytes stimulates lipolysis by activating beta-adrenergic receptor 3 (β3-AR) [6], leading to the release of free fatty acids into the bloodstream to meet whole-body energy demands (Figure 1).

On the contrary, brown adipose tissue (BAT) is implicated in thermoregulation and acts as a metabolic waste for excess lipids through the activation of energy expenditure by non-shivering thermogenesis. In addition, BAT exerts a beneficial impact on whole-body metabolism, particularly on insulin resistance and glucose homeostasis [7]. BAT depots are located in interscapular regions in infants and young children and are detectable in cervical and supraclavicular regions in 3–7% of adults [8,9], and tend to be reduced in obese and elderly subjects [8,10]. Under cold stimulation, thermogenesis in rodents has been shown to result from the sympathetic delivery of noradrenaline to brown adipocytes, activating membrane receptor β3-AR coupled to Gα_S_ and leading to the activation of Protein Kinase A (PKA) through adenylate cyclase activation and the intracellular production of cAMP [11]. The PKA signaling pathway enhances lipolysis via the activation of ATGL and other lipases and increases mitochondrial biogenesis and the activation and upregulation of uncoupling protein 1 (UCP1), which is responsible for mitochondrial uncoupling [12]. In humans, the importance of β3-AR remains debated, with recent works attributing the thermogenic effect to other beta-adrenergic receptors, particularly to β2-AR [13,14], which could explain the poor BAT activation achieved by selective β3-AR agonists [15], while other work confirmed the importance of β3-AR for thermogenic processes [16] (Figure 1).

Fairly recently identified, beige adipocytes are interspersed in white adipose depots [17] but have a thermogenic capacity contrary to that of white adipocytes [18]. Differentiation into beige adipocytes (the so-called beiging process) has been demonstrated to be induced by β3-AR stimulation in rodent models via the action of the transcription factor Prdm16 [19,20]. Recent works have demonstrated that beige adipocytes do not achieve thermogenesis uniquely through mitochondrial uncoupling via UCP1. A strong thermogenic capacity was attributed to futile Ca^2+^ cycling induced by SERCA2b, governed similarly by adenylate cyclase, with β3-AR activation accounting for 70% and α1-AR activation accounting for 30% [21] (Figure 1). Interestingly, this process in beige adipocytes is fueled by increased glycolysis and glucose oxidation, which might participate in the beneficial effect on the global metabolic status observed under beige adipocyte stimulation, particularly the improvement in glucose handling [15,19,20,21].

#### 2.1.2. Endocrine Role in Cardiovascular Setting

The accumulated knowledge of these different depots and types of adipocytes and their biology has led to the recognition of the major endocrine function of adipose tissues. As one of the largest endocrine organs, adipose tissue modulates whole-body metabolism and very diverse processes through paracrine and endocrine actions on distant organs, as initially observed in the liver, pancreas, autonomic nervous system and skeletal muscle [22]. To cite a few, leptin, predominantly secreted by white adipocytes, acts on receptors in the hypothalamus to regulate appetite and improve peripheral insulin resistance [23,24]. Neuregulin 4 (NRG4) is released from thermogenic brown and beige adipocytes, lowers de novo lipogenesis in hepatocytes and prevents liver damage [25]. For a more complete overview, see [22].

Among the diverse adipokines, several affect cardiovascular function via endocrine and paracrine actions. Adipocyte-derived adiponectin is crucial for the maintenance of global glucose homeostasis and insulin sensitivity [26,27]. The circulating levels of adiponectin are downregulated in obesity and contribute to cardiometabolic diseases [28,29], in part due to its protective effect on pancreatic β-cells [30] and on the sympathetic nervous system response, thereby preventing cardiac sympathetic remodeling and cardiac remodeling from occurring upon myocardial infarction [31]. In addition, in cardiac myocytes, adiponectin was found to decrease NADPH oxidase (NOX) activity, thereby exerting an antioxidant effect, an effect possible as a result of both endocrine communication from distant adipose tissue and paracrine communication from epicardial adipose tissue (EAT) [32], as EAT surrounding the myocardium and coronary arteries is in direct contact with cardiac myocytes and adventitia. In this regard, the EAT pathological stimulation observed in coronary insufficiency [33] induces atrial fibrosis via paracrine adipofibrokine signaling, particularly activin A [34]. In addition, chronic atrial fibrillation (AF) has been associated with EAT enlargement and, more recently, EAT remodeling [35,36,37] (see Section 4.3).

As mentioned in the above section, the detrimental effect of visceral adipose tissue on cardiac aging is mediated by secreted adipofibrokines such as osteopontin, leading to the stimulation of myocardial fibrosis through the modulation of fibroblast senescence [5]. On the contrary, the lipokine 12,13-dihydroxy-(9Z)-octadecenoic acid (12,13-diHOME), produced by beige and brown adipocytes and previously shown to increase skeletal muscle fatty acid oxidation [38,39], has been recently associated with improved cardiac hemodynamics through the activation of cardiac nNOS similarly to exercise-mediated cardioprotection, with reduced plasma 12,13-diHOME detected in patients with heart disease [40].

### 2.2. Paracrine and Endocrine Communication by Extracellular Vesicles

#### 2.2.1. Importance of Extracellular Vesicles in Adipose Tissue Cross-Talk

Over the past 10 years, research has uncovered a wealth of intercellular and interorgan signals mediated by small lipid-derived nanovesicles known as extracellular vesicles (EVs). EVs can contain a wide range of bioactive molecules, such as nucleic acids, proteins, lipids or cellular components. All cell types can release EVs that deliver cargo to adjacent cells or even distant organs. However, recent studies have shown that, in the case of adipose tissue, EVs constitute an essential part of the human adipose secretome [41,42].

Mature beige, brown and white adipocytes have all been shown to secrete EVs, which is also the case for adipose-derived stem cells (ADSCs) [43,44]. While the entirety of the adipose-EV secretome remains poorly understood, it has already become clear that the released contents embedded in EVs vary depending on the type of adipocyte and metabolic state and differentially affect the receiving cells, as recently demonstrated by Jung and collaborators after treating diet-induced obese mice with EVs derived from white or beige adipocytes, where the amelioration of hepatic steatosis and glucose tolerance was observed in animals receiving beige-adipocyte-derived EVs [45]. In this regard, beige adipocytes have been previously shown to be an important releaser of EVs, with an 11-fold increase in EV production in response to cAMP [43].

Of note, other cell types present in adipose tissues, such as endothelial cells, have been demonstrated to transfer cargo proteins to adipocytes by EVs such as Caveolin 1, as observed in an adipocyte-specific Caveolin 1 knock-out mouse model [46]. Interestingly, this transfer appears to be affected by metabolic status, as EV secretion from endothelial cells increased in fasting conditions through glucagon signaling [46], while obesogenic diets blocked this effect.

Using an adipocyte-specific Dicer knock-out mouse model, Thomou and collaborators demonstrated that the majority of circulating miRNAs transported by EVs actually derive from adipocytes [47], suggesting that adipocyte-derived EVs (AdEVs) represent the majority of circulating EVs. In addition, AdEVs have been shown to reach and be taken up by cardiac tissues, as observed many times using the fluorescent labeling of adipose-derived EVs [48,49].

#### 2.2.2. Type of Extracellular Vesicles and Biogenesis

EVs, described as particles delimited by a lipid bilayer that are naturally released from the cell and cannot replicate, are classically divided into three categories (exosomes, microvesicles and apoptotic bodies) mainly based on their size and biogenesis [50].

Small EVs called exosomes with sizes ranging from 50 nm to 100–150 nm are generated via the endocytic pathway. Exosomes are formed as intraluminal vesicles (ILVs) within the lumen of multivesicular endosomes (MVEs). Thereafter, MVE fusion with the plasma membrane enables the release of exosomes into the extracellular space.

Larger microvesicles (100 nm up to 1 µm) [50] are formed directly via budding/blebbing of the plasma membrane. Here, the rearrangement of lipid and protein composition at the plasma membrane, as well as changes in local Ca^2+^ levels, takes place. The activation of Ca^2+^-dependent enzymatic machineries such as aminophospholipid translocases, scramblases and calpain drives the rearrangement of phospholipids at the cell membrane, notably the exposure of phosphatidylserine from the inner leaflet to the cell surface, which causes the physical outward bending of the membrane. Together with the rearrangement of the underlying actin cytoskeleton, this process favors membrane budding and the formation of microvesicles [50,51].

As for apoptotic bodies (1 to 5 µm), they are specifically released by cells undergoing apoptosis. Here, the activation of programmed cell death triggers plasma membrane blebbing and nuclear fragmentation [52]. Apoptotic bodies in the extracellular space can then be removed by phagocytic cells to prevent damage to adjacent cells. To date, their implications in cell–cell communication have been assumed to be less important than those of microvesicles and exosomal communication and have been, therefore, poorly investigated.

Nevertheless, EVs represent a heterogeneous population, and at this stage in our understanding of EV biology, some features may overlap between categories. As a matter of fact, there are currently no known markers specifically defining each vesicle subtype. Therefore, the International Society for Extracellular Vesicles (ISEV) recommends the use of the generic term “extracellular vesicle” [50,53].

## 3. Adipose Tissue Diversity and Remodeling: EV-Mediated Influence on the Heart

### 3.1. Brown Adipose Tissue and Beige Adipocytes

**Figure 2:**
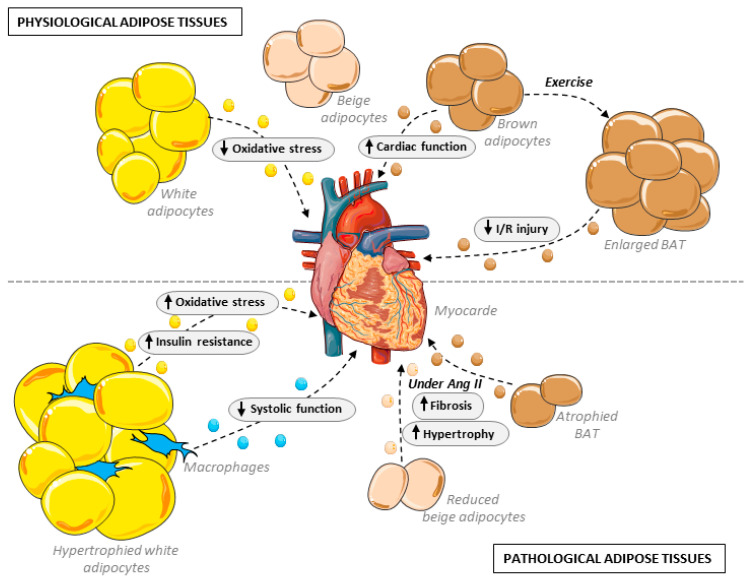
Schematic representation of the impact of adipose-derived EVs on heart function in healthy and pathological adipose tissues as seen in the context of obesity.

In the last several years, light has been shed on the importance of AdEVs secreted by BAT in the preservation of cardiovascular physiology. Earlier this year, Zhao et al. demonstrated that small AdEVs secreted by brown adipose tissues play an important part in the cardioprotection induced by exercise. In their study, a period of exercise of 4 weeks was sufficient to induce the enlargement of BAT, and the corresponding secreted EVs were able to attenuate reperfusion injury following myocardial infarction (Figure 2). Cross-comparison using RNAseq of circulating EVs and BAT-secreted EVs in sedentary rodents or upon exercise identified miR125-5p, miR-128-3p and miR-30d-5p as essential for the observed cardioprotection by lowering apoptosis induced by the MAPK pathway [54]. Similarly, Lin et al. demonstrated a cardioprotective effect mediated by EV communication from BAT to cardiac myocytes and cardiac fibroblasts. Here, the authors showed that β3-AR deletion in UCP1-expressing cells by crossing *UCP1*-Cre^+/−^ with *adrb3*^flox/flox^ animals attenuated BAT thermogenesis and accelerated angiotensin II-induced cardiac remodeling (Figure 2). On the contrary, treatment with exosomes from brown adipocytes treated with a β3-AR agonist conferred protection against AngII-induced cardiac remodeling, although, here, a high dosage of EVs via injection was used (4 mg/kg). The study further pinpointed iNOS as a critical cargo component of EVs derived from beige adipocytes with β3-AR knockdown and contributing to cardiac fibroblast dysfunction and cardiac remodeling, and its secretion by BAT EVs was reduced under BAT stimulation [49]. It should, however, be noted that while the study mentioned only brown adipocytes, the cardiac remodeling observed in AngII-treated animals harboring *UCP1*- Cre^+/−^ combined with *adrb3*^flox/flox^ could well be a combined effect of the dysfunction of both brown and beige adipocytes and not only BAT, as beige adipocytes do express UCP1 to a significant degree. In addition, the recent comparison of lncRNA in EVs from WAT and BAT and the identification of over 500 differentially expressed lncRNAs [55] might lead in the coming years to the uncovering of lncRNAs additionally involved in the cardioprotection mediated by EVs secreted by brown adipocytes.

While the studies mentioned above highlight the cardiovascular protective action of brown-adipocyte-derived EVs, deleterious effects were observed upon in vitro treatment with rosiglitazone. Rosiglitazone, a peroxisome proliferator-activated receptor-gamma (PPARγ) activator, is used as a treatment for type II diabetes and is a well-known in vitro inducer of browning in adipose mesenchymal stem cells (ADSCs), and it has recently been found to trigger the adipocyte-specific release of circulating miR-200a embedded in EVs. miR-200a induces cardiac hypertrophy through the downregulation of TSC1, thus relieving its inhibition of mammalian target of rapamycin (mTOR) signaling [56]. These findings provide an explanation for the cardiac hypertrophy observed in pre-clinical models upon treatment with rosiglitazone (and PPARγ agonists in general) without affecting cardiac PPARγ and possibly for the increased cardiovascular risk observed in clinical trials, particularly heart failure, associated with rosiglitazone treatment that led, depending on the country, to drug bans or restrictions for prescription [57]. As PPARγ is a key regulator of brown adipocyte differentiation [58], browning agents should therefore be carefully screened for the similar secretion of miR-200a-containing EVs before moving toward implementation in the clinic.

### 3.2. Cardiovascular Effects through Vascular Implications

Extracellular vesicles derived from perivascular adipose tissue (PVAT) have been shown to be implicated in the important roles of PVAT related to the regulation of vascular physiology and pathophysiology. In endothelial cells, protection against oxidative stress and atherosclerosis has been demonstrated by restraining miRNA-342-5p expression in EVs derived from adipose mesenchymal stem cells (ADSCs) [59]. Recent work has shown that EVs derived from PVAT protect against atherosclerosis through an EV-mediated reduction in macrophage foam cell formation. In particular, miR-382-5p secreted by PVAT in EVs has been identified to upregulate cholesterol transport proteins in target macrophages, thereby reducing macrophage lipid accumulation and foam cell formation [60,61]. In addition, lower levels of miR-382-5p were detected in EVs from the PVAT of coronary atherosclerotic heart disease patients compared to healthy individuals [60]. On the contrary, EVs isolated from visceral adipose tissues produced the opposite effect, increasing macrophage foam cell formation, although the implicated factors targeting macrophages here have not yet been identified [62].

An additional microRNA, miR-221-3p, found in EVs secreted by PVAT has been shown to participate in the detrimental vascular remodeling that occurs with a high-fat diet [63]. In the conditions of low-grade inflammation in PVAT, miR-221-3p led to a phenotypic switch from a contractile to a synthetic phenotype in the aorta and possibly early-stage vascular dysfunction due to the observed reduction in contractile gene expression levels in VSMC. It remains, however, unclear which cell types within PVAT are responsible for the enrichment of EVs with miR-221-3p in the context of obesity.

In obesogenic conditions, EVs from visceral adipose tissues also affect the vascular beds by inducing the M1 proinflammatory polarization of macrophages and thus exacerbating the progression of atherosclerosis [62]. In a recent study, the thoracic visceral AT volume (corresponding here to peri/epicardial AT, although not mentioned verbatim) was significantly correlated with arterial oxidative stress [64]. Focusing on dysregulated metabolites, Akawi and collaborators identified the high thoracic AT secretion of sphingolipids, associated with reduced endothelial NO bioavailability and increased superoxide in vessel biopsies. The ceramide Cer16:0 in particular was significantly associated with oxidative stress. Further investigations revealed that in vitro treatment with Cer16:0, being measured as the most abundant ceramide generated in thoracic AT and secreted largely in extracellular vesicles, increased ROS production by eNOS uncoupling and protein phosphatase 2 dysregulation in human aortic endothelial cells. Importantly, ceramides are directly implicated in EV microvesicle formation through ceramide-rich membrane patches [50], and it remains unclear whether these correlations with endothelial oxidative stress are linked to free Cer16:0 circulating as the cargo of EVs, in which case ceramides may act on intracellular signaling, as observed in vitro by Akawi et al., or linked to membrane-rich Cer16:0 levels, in which case the association with oxidative stress could be instead linked to overall EVs levels. Interestingly, intervention with a glucagon-like peptide-1 analog could suppress plasma Cer16:0 and its derivatives. Since high plasma Cer16:0 has been correlated with an increased risk of cardiac mortality, this could have therapeutic potential.

Finally, modifications in the secretion profile of PVAT-derived EVs have also been observed during pregnancy. Interestingly, these modifications were restricted to uterine PVAT, while the periovarian adipose-tissue-derived EV profile was unchanged. These alterations modified EV morphology and cargo, with smaller vesicles of higher density in pregnant female rats, although the functional consequences for VSMC and vasorelaxation remain to be explored [65].

### 3.3. Adipose Tissue Remodeling and Cardiac Consequences

#### 3.3.1. Aging

EVs from adipose mesenchymal stem cells (ADSCs) could possibly play a protective role against aging-induced cardiac dysfunction. In a recent study, the authors observed that treating old mice with EVs derived from the ADSCs of young animals improved several parameters classically altered with aging, such as fatigue resistance, renal function and motor coordination [66]. Since these improvements were associated with reduced oxidative stress, inflammation and senescence markers in muscle and kidney, similar effects could likely participate in the protection of the heart and vasculature during youth, and their absence could be implicated in cardiac pathologies affecting older populations, in which inflammation and oxidative stress are implicated in conditions such as heart failure with preserved ejection fraction [67,68]. Further work will be needed to determine which cargos in EVs derived from the ADSCs of young animals are responsible for this effect and whether these cargos could prevent or delay heart failure progression.

In addition to the modification of the ADSC secretome, aging affects brown adipose tissue, leading to a decrease in its function and thermogenic capacity [8,9,69]. Therefore, cardioprotective actions mediated by the BAT-derived EVs mentioned above (Section 3.1) would most likely be lost during aging. In such conditions, the heart would be more prone to stress-induced cardiac remodeling. To some extent, the deletion of β3-AR in UCP1-expressing cells, as shown in a study carried out by Lin et al., mimics the loss of BAT stimulation that progressively occurs during aging [49], where the loss of thermogenic capacity is paralleled with accelerated angiotensin II-induced cardiac remodeling due to an increased level of iNOS transported by EVs from the dysfunctional BAT to cardiac myocytes and fibroblasts. This effect could well contribute to the worsening of cardiac pathologies and the worse outcomes observed in older patients.

#### 3.3.2. Obesity

Western diets containing high levels of saturated fatty acids and sugars, generating an overload of circulating nutrients, induce the hyperplasic and hypertrophic remodeling of white adipose tissue (WAT), leading to inflammation and obesity and durably altering WAT endocrine communication. In this regard, WAT-derived EVs have been shown to be altered in metabolic diseases [70,71].

Long-term Western or high-fat diets (HFDs) have been associated with cardiac injuries [72,73]. Earlier this year, Zhao and collaborators provided evidence that EVs derived from the adipose tissue macrophages of obese mice on an HFD provoked cardiac injuries associated with systolic dysfunction compared to EVs from lean mice (Figure 2). This observation was linked to an increase in cardiac lipid peroxides and mitochondrial damage on an HFD. The authors further identified miR-140-5p as a cargo enriched in macrophage-derived EVs on an obesogenic diet and demonstrated that miR-140-5p promotes ferroptosis in cardiomyocytes (i.e., cell death caused by excessive iron-dependent lipid peroxidation) by regulating GSH synthesis [74].

Several additional miRNAs transported by EVs have been highlighted as enriched in plasma under obesogenic conditions and implicated in obesity-related cardiomyopathy. In this regard, miR-194 and miR-29a were found to be closely correlated with cardiac dysfunction, including ejection fraction and the biomarker NT-proBNP in obese patients [75,76]. Both miRNAs impaired cardiac mitochondrial activity, but the underlying mechanisms remain to be uncovered, as well as the origin of these EVs (WAT, BAT, macrophages or hepatocytes). In addition, miR-802-5p, identified for its enrichment in EVs- from white adipocytes in obesogenic conditions, has been shown to induce insulin resistance and oxidative stress in cardiac myocytes through the downregulation of HSP60 [77].

In addition to microRNA, while others have previously shown that adipocyte-derived EVs could transport mitochondrial proteins and components affecting metabolism to different organs [46,78,79,80,81], recent work by Crewe and collaborators evidenced the release of EVs containing stressed but functional mitochondrial particles into the bloodstream by adipocytes in obesogenic conditions. These EVs are taken up by cardiomyocytes, where they trigger compensatory antioxidant signaling that acts as ischemic preconditioning and attenuates cardiac ischemia/reperfusion injury [48].

Human antigen R (HuR) is a member of the Hu family of RNA-binding proteins. HuR expression in subcutaneous WAT has been inversely correlated with obesity in human patients and appears to be critically involved in the regulation of lipolysis via ATGL expression, thereby having clinical implications in metabolic syndrome [82]. Recently, Guarnieri et al. demonstrated that adipose-specific deletion leads to cardiac hypertrophy and fibrosis. Data obtained by RNAseq revealed an inflammatory response in both adipose and cardiac tissues, as well as an enrichment in genes related to exosome formation/secretion in adipose depots that correlated with the degree of fibrosis severity [83]. This finding warrants further investigations to understand the extent to which HuR is implicated in EV formation in WAT.

A reduction in or the complete loss of brown adipose tissue is observed in patients with overweight or obesity [8,9,84]; therefore, similar to aging, the loss of cardioprotective effects due to a reduction in brown adipocytes is likely. As a matter of fact, the intravenous administration of exosomes isolated from brown adipose tissue for six weeks led to improved cardiac function and the amelioration of metabolic syndrome in mice on an HFD [85]. This might well be due to the direct effect of EVs on the heart, although beneficial effects could in part be attributed to the normalization of glucose handling and reduced obesity.

Importantly, major adipocytokines that are implicated in antioxidant effects, anti-inflammatory activity and insulin sensitivity in the cardiovascular system and are observed to circulate at lower levels in obesity, such as omentin-1 and adiponectin [86,87,88], have been reported to be enclosed in EVs derived from WAT [89]. Omentin-1 was associated mostly with EVs from visceral AT, while adiponectin could also be found in EVs from subcutaneous AT tissue, but only for high- and medium-molecular-weight adiponectin [89]. As the reduced circulation of anti-inflammatory adipocytokines has been associated with a higher risk of cardiovascular diseases [90], further studies are warranted to determine to what extent EVs are implicated in adipocytokine protection or contribute to obesity-induced cardiac damage.

## 4. Adipose-Derived EVs in the Pathological Heart

### 4.1. Cardiac Remodeling

In the context of neurohormonal or hemodynamic stress, the adverse remodeling of the myocardium occurs, characterized by cardiac myocyte hypertrophy and fibrosis development, with its progression being a determinant of the onset of heart failure. During this remodeling, EVs derived from adipose tissue have been found to positively or negatively affect cardiac function and remodeling. To date, very little of the great diversity of EVs has been investigated in relation to the remodeling of the heart.

Recent work has evidenced the worsening action of EVs derived from visceral adipose tissue on cardiac function and fibrosis in angiotensin II-infused mice [91]. Enrichment in miR-23a-3p identified in EVs derived from Ang II-challenged adipocytes and serum EVs from Ang II-infused mice was demonstrated to cause cardiac fibrosis and dysfunction. Mechanistically, miR-23a-3p would accelerate excessive collagen deposition by acting on its downstream target RAP1, a GTPase recently shown to be involved in cardiac fibroblast transition into myofibroblasts and collagen deposition [92]. This was further confirmed by the overexpression of RAP1, during which the profibrotic effect of miR-23a-3p was reversed [91].

In similar conditions of AngII infusion, the deletion of β3-AR from brown and beige adipocytes led to the worsening of cardiac function as well as cardiac fibrosis and hypertrophy, as previously mentioned (see Section 3.1). This effect was attributed to brown-adipocyte-derived EVs taken up by cardiac myocytes and cardiac fibroblasts and, in particular, to the enrichment of iNOS in EVs upon β3-AR deletion [49]. It is still unclear whether iNOS expression and its exosomal release are specifically regulated by β3-AR or are a side effect of brown adipocyte dysfunction due to β3-AR knock-out. Nonetheless, this could well mimic, as mentioned in the above section, the loss of BAT stimulation observed in both aging and obesity. Additionally, interscapular brown adipose depots are undetectable in a large part of the adult population (detectable in 5% of adults > 30 years old), for which it is unclear whether smaller brown adipocytes depots interspersed in white adipose depots could still be present and functional [8,9]. This could therefore contribute to explaining the worsening of cardiac pathologies in certain patient populations.

In the past several years, EVs derived from ADSCs have gained considerable interest as a cell-free therapeutic alternative and as a potential vehicle to deliver bioactive factors due to their relatively easy isolation and propagation in culture. This interest derives in part from the cardioprotective effects observed with EVs from ADSCs during cardiac ischemic events (see Section 4.3). Moreover, the protective effects of ADSC-derived EVs against cardiac remodeling have also been reported. When facing hemodynamic stress, the elevation of adiponectin plasma levels reinforced the cardioprotective action of injected ADSCs by significantly increasing EV release, as observed in a mouse model of pressure overload. This increase in EV release further improves the amelioration of ejection fraction, fractional shortening and heart weight observed upon ADSCs injection alone [93]. A similar cardioprotective effect of ADSC-derived EVs under neurohormonal stress was later confirmed, causing a reduction in the hypertrophic response and proinflammatory signaling imposed by angiotensin II and TGFβ1 treatment on human-induced pluripotent-stem-cell-derived cardiomyocytes [94]. The intrarenal delivery of EVs from ADSCs has also been reported to attenuate kidney inflammation and renal artery stenosis (RAS) in a swine model of RAS and metabolic syndrome via anti-inflammatory pathways [95]. Importantly, such treatment also blunted secondary myocardial injury, possibly through the improvement of both renal function and systemic inflammation [95]. Earlier this year, this effect was attributed to EVs’ anti-inflammatory IL-10 cargo decreasing activated T-cell proliferation and endothelial inflammation [96].

### 4.2. Diabetes and Diabetic Cardiomyopathy

Type II diabetes is classically associated with dysfunctional and senescent white adipocytes [97] due to the increased inflammatory state and profound alterations in systemic metabolism, such as impaired glucose handling. EVs derived from dysfunctional visceral white adipocytes in obesogenic conditions (i.e., high-fat diet) have been described as responsible for the exacerbation of myocardial ischemia/reperfusion (MI/R) injury observed in diabetic patients compared to healthy subjects [98]. In this regard, Gan et al. identified the elevation of miR-130b-3p in adipose-derived EVs from diabetic rats and in the plasma of diabetic patients as causative of this aggravation via the induction of the cardiac myocyte proapoptotic response [98].

Dysfunctional and senescent adipocytes have also been implicated in the pathogenesis of diabetic cardiomyopathy [97]. In their study, Lin et al. evidenced that the removal of epididymal adipose tissue (visceral WAT) could alleviate the diastolic dysfunction observed in streptozotocin (STZ) mice [97] and that EVs from these adipose depots drive contractile (return velocity and shortening) and mitochondrial dysfunction in isolated cardiac myocytes. The authors identified upregulated miRNA-326-3p in EVs derived from senescent adipocytes and its inhibition of Rictor expression as responsible for these cardiac impairments. In addition, miR-802-5p enriched in EVs from cultured hypertrophic adipocytes has been reported to contribute to cardiac insulin resistance [77], most likely through the downregulation of HSP60, which has been shown to regulate Insulin-like Growth Factor-1 Receptor (IGF-1R) signaling and be inversely correlated with diabetic cardiomyopathy [99].

### 4.3. Epicardial Adipose Tissue and Atrial Fibrosis and Fibrillation

Epicardial adipose tissue (EAT) is composed of visceral adipocyte depots distributed between the myocardium and the epicardium and surrounding the coronary arteries and the myocardium [100].

EAT has been demonstrated to contribute to the development of atrial fibrillation (AT). The enlargement of EAT depots is associated with AF [35,36,37], and nowadays, the volume of atrial EAT is considered to be a determinant of the risk and persistence of AF. More recently, the state of remodeling of atrial EAT was also implicated in the development of AF [37]. Given their anatomical proximity, profibrotic factors (such as matrix metalloproteinases and activin A) and proinflammatory factors (such as interleukins and TNF) released by EAT can easily reach the atrium, leading to atrial myocardial fibrosis [101]. In addition, the increased influx of free fatty acids from atrial EAT could also cause conduction abnormalities [102]. Altogether, the paracrine communication of atrial EAT is believed to be responsible for the arrhythmogenic effects of atrial EAT on cardiomyocytes. A recent study performed on EAT from patients suffering from AF or other heart diseases pointed out the critical role of EVs in atrial regulation, as EVs from both types of patients led to a shortening of the action potential duration in induced pluripotent-stem-cell-derived cardiac myocytes, which predisposes cardiac myocytes to AF [103]. Interestingly, EVs isolated from the EAT of patients suffering from AF led to elevated proliferation levels of stromal and endothelial cells and increased the propensity for reentrant arrhythmia compared to patients with other heart diseases [103]. The exact factors implicated here are not yet pinpointed, but the molecular and proteomic investigation of the cargos of these particular EVs identified the enrichment of cytoskeletal regulation, inflammatory, fibrotic and angiogenic proteins, as well as miRNAs implicated in the pro-fibrotic response (such as miR-146b) [103].

In coronary insufficiency, the pathological stimulation of EAT surrounding the coronary arteries has been shown to induce atrial fibrosis [33] via adipofibrokine paracrine signaling, particularly activin A [34], further contributing to the development of coronary artery disease (CAD). To date, the exact mode of transfer of these adipofibrokines in CAD has not yet been investigated but could well involve extracellular vesicles, as observed in the case of atrial fibrillation.

### 4.4. Ischemia/Reperfusion

Research in the last five years has produced a body of evidence in favor of a cardioprotective effect of EVs derived from healthy ADSCs against ischemic myocardial injury; details are summarized in Table 1, with the identified cargos and corresponding regulated pathways in myocytes (Table 1). In this regard, EVs from ADSCs could be useful for a therapeutic approach to acute cardiovascular ischemic events. Interestingly, adiponectin seems to effectively stimulate AdEV release from ADSCs and reinforce cardioprotective action [93,104].

In healthy adult adipocytes, miR-30d, miR-125b and miR-128 delivered to the heart by EVs released from brown adipocytes upon exercise have been shown to lower apoptosis induced by the MAPK pathway and therefore exert a cardioprotective action against ischemic myocardial injury [54]. Conversely, small EVs released from the ischemic heart were recently shown to target and enter white adipocytes, causing adipocyte dysfunction. The underlying mechanism involves miR-27a transfer by EVs to adipocytes and the subsequent downregulation of EDEM3 (ER degradation-enhancing alpha-mannosidase-like protein 3), leading to adipocyte ER stress [105].

**Table 1:** miRNAs and circRNAs enriched in EVs derived from healthy ADSCs and their cardioprotective effects against ischemic myocardial injury (MI/R: myocardial infarction/reperfusion; H/R: hypoxia/reperfusion; ↑ or ↓ for increased or decreased processes).

Species	MI Model	Identified/Implicated Cargo	Effect on Cardiac Function and Cardiac Myocytes	Effect on Cardiac Fibroblasts	Effects on Other Cell Types	Reference
Rat	MI/R	miR-425-5	↓ mitochondrial dysfunction ↓ ROS production	-	↑ angiogenesis ↑ M2 macrophage polarization	[106]
		miR-196a-5p	↓ mitochondrial dysfunction ↓ ROS production	↓ myofibroblast activation	↑ angiogenesis ↑ M2 macrophage polarization	[106]
		miR-146a	↓ reduced apoptosis	-	-	[107]
		miR-126	↓ proinflammatory cytokine	↓ fibrosis	↑ angiogenesis	[108]
		miR-93-5p	↓ hypoxia-induced autophagy ↓ inflammatory cytokine expression by targeting Atg7 and TLR4	-	-	[109]
Mouse	MI/R	miR-221/222	↓ PUMA/ETS-1 pathway ↓ H2O2-induced cell apoptosis	-	-	[110]
		miR-214	↓ reduced apoptosis ↓ EGR1 and hypoxia-induced TLR4/NFκB	↓ fibrosis	-	[111]
		miR-210	↑ mitochondrial bioenergetics ↓ ROS production	-	-	[112]
		miR-31	-	-	↑ angiogenesis ↑ endothelial cell migration ↑ tube formation Target FIH1 and ↑ HIF-1α activation	[113]
	MI/R anoxic preconditioning of ADSCs	miRNA224-5p	↓ reduced infarct size ↓ reduced apoptosis TXNIP downregulation	-	-	[114]
Pig	MI/R	-	↓ end-diastolic pressure–volume ↓ CM hypertrophy	-	-	[115]
		-	↑ contractile function	-	↑ angiogenesis ↑ myocardial perfusion	[116]
Primary cells	H/R in NMCM	miR-671	↓ TGFBR2/Smad2 pathway ↓ reduced apoptosis	-	-	[117]
	H/R in NRCM	-	↓ reduced apoptosis ↑ VEGF, bFGF, HGF	-	-	[118]
Cell lines	H/R on HL-1	circ_0001747	Sequestration of miRNA-199b-3p ↑ MCL1 pathway	-	-	[119]
	H/R on H9c2	-	↑ S1P/SK1/S1PR1 signaling	-	↑ M2 macrophage polarization	[120]

As for dysfunctional adipocytes, the influence of their derived EVs on cardiac injury is still debated, with evidence, as mentioned in Section 4.2, of the exacerbation of MI/R injury observed in diabetic patients attributed to the elevation of miR-130b-3p in EVs and the subsequent induction of the cardiac myocyte proapoptotic response [98]. On the contrary, others have observed the uptake by cardiac myocytes of large EVs containing stressed but functional mitochondrial particles derived from adipocytes subjected to obesogenic conditions. These oxidatively stressed mitochondria activate compensatory antioxidant pathways in cardiac myocytes, thereby protecting against I/R damage, as does ischemic preconditioning [48].

## 5. Conclusions

Recent research on the biology of EVs derived from adipose tissue has provided us with a better understanding of the modulation that these EVs can exert on cardiac physiology, whether protective or detrimental. The technical advances in isolation and purification in this exciting and emerging area of research have enabled the scientific community to gain a better understanding of the roles played by AdEVs. These advances remain particularly important to decipher whether endocrine factors secreted by adipose tissue, already described as affecting the heart, are mainly transported via EVs in physiological or pathological conditions, as major adipokines such as omentin and adiponectin have been reported in circulating EVs.

It is now established that a wide diversity of signals (miRNA, proteins, lncRNAs, adipokines, enzymes, etc.) are released via AdEVs and are taken up by cardiac myocytes and other resident cells of the myocardium (fibroblasts, endothelial cells, etc.). Here, technical advances could also help identify, in a faster and more rigorous manner, the factors implicated in the observed effects, particularly through the isolation of specific subpopulations of EVs based on size (exosomes vs. microvesicles) or origin, using specific markers of EVs derived from mature adipocytes, ADSCs or immune cells.

Depending on the cardiac pathology or the type of cardiac stressor, AdEVs may have very different impacts. This is also found to be true with regard to adipose tissue, which has a striking biological variability depending on its metabolic status and location (functionality of brown adipocytes and levels of ADSCs in aging, EAT vs. other depots, etc.) and affects the overall cardiometabolic risk of each individual. Therefore, significantly more work is needed to gain a clearer understanding of the effects of dietary status, age and overall adipose tissue physiology or pathophysiology on specific cardiac conditions, particularly the impact of AdEV subpopulations on cardiac myocyte metabolism and contractility, two crucial aspects of cardiac function that have yet to be investigated. In addition, sex and gender differences have been observed in both cardiovascular risks and adiposity patterns (roughly summarized, more SAT deposition in females and more VAT in males). Here, the implications and causality of EVs should be explored.

Finally, while therapeutic EV delivery offers many advantages, such as rapid clearance, low immunogenicity and non-cytotoxicity when derived from host cells, this will most likely not be true for EVs derived from different biological origins than the host. The process of the inclusion of cytoplasmic and membrane proteins within EVs is still unclear and leads to a wide variety and heterogeneity of embedded proteins, which would likely trigger a significant immune response. In addition, the process of EV modification and EV administration should be investigated with caution, as EVs may well be internalized by several cell types, which could lead to unpredictable side effects. These aspects should be carefully explored in order to fully dissect the potential therapeutic use of AdEVs.
